# Screening Amazon rainforest plant extracts for antimicrobial activity: a 15-year commitment to the Brazilian biodiversity

**DOI:** 10.3389/frabi.2023.1122400

**Published:** 2023-07-12

**Authors:** Ivana Barbosa Suffredini, Jefferson de Souza Silva, Sergio Alexandre Frana, Katia Cristina Pinto, Keli Cristina Dias Bento, Erika Costa Rudiger, Paloma Kelly de Souza Belo, José Rodrigo de Arruda, Juliana Paola Schulze, Adriana Lígia de Castilho, Livia Roberta Piedade Camargo, Ricardo Olivieri Paulino, Yasmin de Oliveira Santos, Raphael Assis Leandro Morais, Karen Cristina Comin Maldonado, Gabriele Kolndorfer, Karolayne da Silva, Pietra Dantas de Jesus, Gabriella de Oliveira Moura, Victoria Rocha Brandão, Hevelton Araújo Ribeiro, Christian Henrique Komka Vara, Fabiane Massola, Ingrit Elida Collantes Díaz, Mateus Luís Barradas Paciencia, Selene Dall'Acqua Coutinho, Riad Naim Younes, Antonio Drauzio Varella

**Affiliations:** ^1^ Center for Research in Biodiversity, Graduate Program in Environmental and Experimental Pathology, Paulista University, São Paulo, SP, Brazil; ^2^ Science Health Institute, Undergraduate Vice-Dean Office, Paulista University, São Paulo, SP, Brazil; ^3^ Chemistry and Textile Engineering Faculty, National University of Engineering, Lima, Peru; ^4^ Hospital Alemão Oswaldo Cruz, São Paulo, SP, Brazil

**Keywords:** Amazon rainforest, large-scale screening, bioautography, susceptibility assay, toxicity assay, thin-layer chromatography

## Abstract

**Introduction:**

The need for new tools to treat infections is constantly growing due to the possibilities of emerging diseases related to environmental changes, climatic catastrophes, microorganism resistance, and human and animal aging, leading to an evident unbalance in the planet’s health. Brazil contains the most significant portion of world biodiversity, a potential source of new antimicrobial natural products. Nonetheless, its environment, particularly its forests, and rainforests, is under threat, meaning that rapidly conducted, comprehensive research into the potential of antimicrobial activity to address this threat is urgently needed.

**Methods:**

In this study, plants from the Amazon rainforest and the Atlantic forests were collected and tested against several pathogenic microbes relevant to humans, animals, and the environment, and subjected to large-scale susceptibility assays, bioautography, and *Artemia salina* toxicity assays. From the plants, 2,280 organic and aqueous extracts were obtained from different organs, namely leaves, barks, flowers, fruits, and seeds, and subjected to a large-scale susceptibility screening assay against *Staphylococcus aureus*, *Staphylococcus epidermidis*, *Enterococcus faecalis*, *Streptococcus mutans*, *Streptococcus sanguinis*, *Escherichia coli*, *Pseudomonas aeruginosa*, *Candida albicans*, *Malassezia pachydermatis*, *Malassezia furfur*, and *Listeria monocytogenes*.

**Results and discussion:**

The selected extracts were subjected to antimicrobial susceptibility tests to determine their inhibition zone diameters and minimum bactericidal concentrations, to bioautography, and to an *Artemia salina* toxicity assay, which resulted in 154 active extracts. Moreover, 111 out of 154 extracts were ranked based on scores established by the *p*-values and the mean rank differences in each set of test results. The final ranking identified which extracts should be studied in further phytochemical research using thin-layer chromatography techniques as a priority. The extracts obtained from plants belonging to Combretaceae, Connaraceae, Convolvulaceae, Fabaceae, Malpighiaceae, Moraceae, Piperaceae, Polygonaceae, and Salicaceae were selected as the most promising ones and used to support the identification of plant-based antimicrobial active compounds from the immense biodiversity of Brazilian forests.

## 1 Introduction

The Amazon rainforest is the largest equatorial forest in the world (Hansen et al., 2013), and the richest biome in terms of biodiversity. The Brazilian Atlantic forest, also a biodiverse biome, has been suffering from the impact of anthropogenic activity for the last 500 years, which resulted in a remaining area of 12.4%. Both biomes must be better understood in terms of their potential as source of new medicines, and how the deforestation can impact on health (SOS Mata Atlântica/ INPE, 2019). The impact of the deforestation causing the resurgence of infectious diseases are well described (Ellwanger et al., 2020). In addition, ecological disturbance leading to alterations in the natural environment of microbes and parasites, and the proliferation, distribution, and consequent survival and reproduction of disease vectors in both forest and urban areas, have been identified as factors contributing to the recrudescence of infectious diseases (Ellwanger et al., 2020).

Infectious diseases have been significant to humans since ancient times. The history of medicine can be told through the view of infectious diseases as they were recorded and interpreted in documents over time, in which plague, syphilis, malaria, smallpox, cholera, dysentery, AIDS, and COVID-19 are the main actors (Sakai and Morimoto, 2022). Infectious diseases also affect wild, domestic, and production animals, resulting in invaluable losses for pet owners and livestock farmers (Peixoto et al., 2021). At the end of the 19th and start of the 20th century, observations that pathogens could be transmitted from animals to humans were made, with several scientific studies considering the inter-relationship between human and animal infectious diseases leading to the concept of zoonosis as we know it (CDC, 2022). Today, it is impossible to separate human health, animal health, and environmental health, for they are intimately connected under the concept of “One World, One Health”, established by Calvin Schwabe in the mid-1960s (Mackenzie and Jeggo, 2019; CDC, 2022). The rational use of antimicrobials and antibiotics mitigates such losses, with new drugs that can target the pathogens causing infectious diseases urgently needed.

In recent decades, our researchers have been systematically developing projects aiming to identify antimicrobial active extracts from the Amazon and Atlantic Brazilian rainforests. We have studied more than 2,000 plant extracts and essential oils as antimicrobials against *Staphylococcus aureus* (Pinto, 2020), *Staphylococcus epidermidis* (Belo, 2022), *Enterococcus faecalis* (Castilho, 2009), *Streptococcus mutans* (Bento, 2020), *Streptococcus sanguinis* (Silva, 2009), *Escherichia coli* (Camargo, 2016), *Pseudomonas aeruginosa*, *Candida albicans* (Rudiger, 2021), *Malassezia pachydermatis* (Silva, 2018), *Malassezia furfur* ^(^ Silva, 2018 ^),^ and *Listeria monocytogenes* (Ceruso et al., 2020), which are shown in the present article as a result of a 15-year study in antimicrobial-active natural product identification (Suffredini et al., 2002; Suffredini et al., 2004; Suffredini et al., 2006a; Suffredini et al., 2006b; Younes et al., 2007; Castilho et al., 2013; Camargo and Suffredini, 2014; Castilho et al., 2014; da Silva et al., 2014; Costa et al., 2016; Suffredini et al., 2016; Martins et al., 2019a; Martins et al., 2019b; Camargo et al., 2020; Silva et al., 2020).. The present article reports a step-by-step methodology for identifying antimicrobial products in nature that comprises plant collection, the different tests used to analyze the *in vitro* antimicrobial activity, a test to determine toxicity, and a statistical-based algorithm to classify the extracts according to their potential. The use of well-established tests allows its reproducibility in different types of pharmacognosy or microbiology laboratories.

## 2 Materials and methods

### 2.1 Plant collection

**Figure 1:**
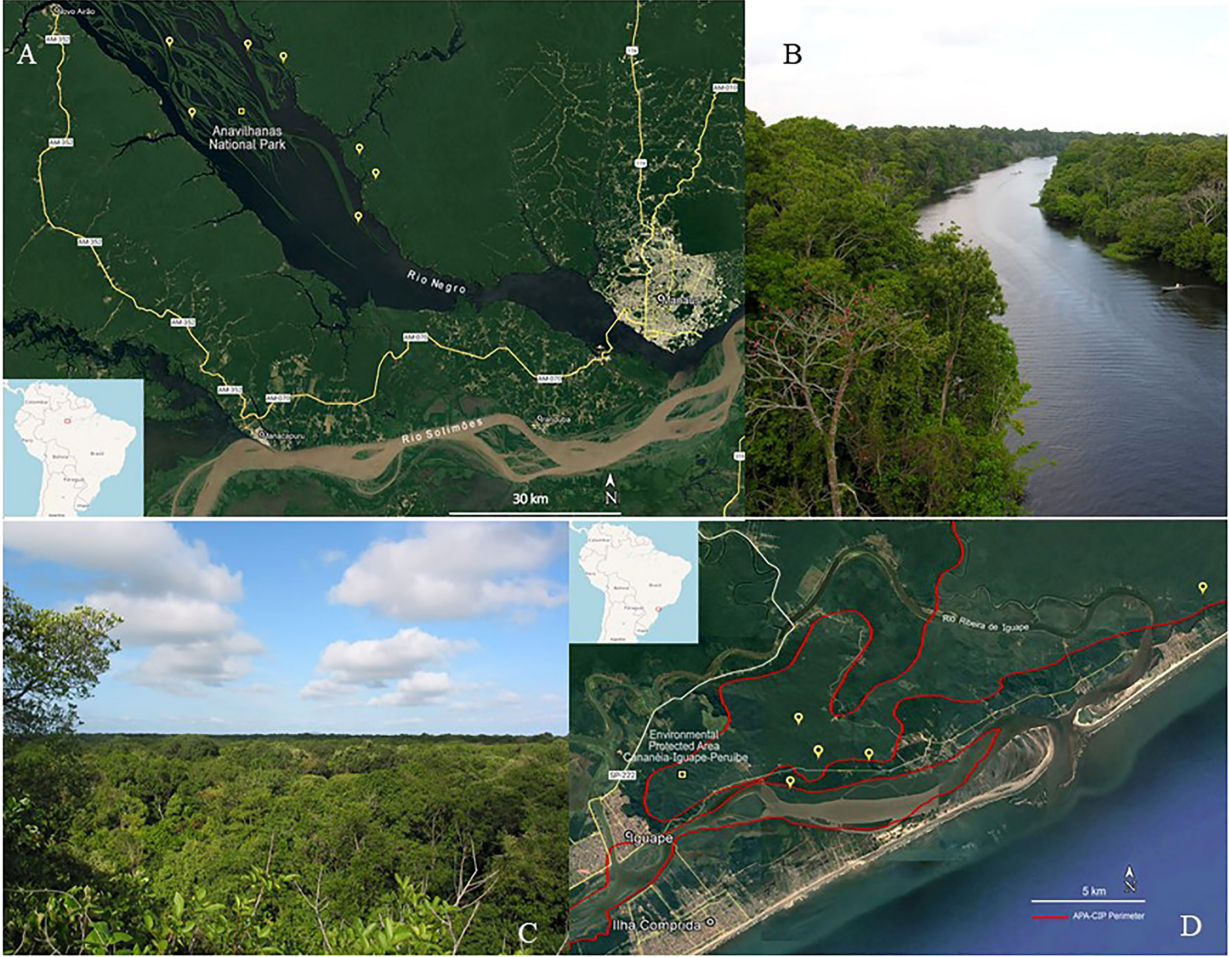
Plant collection. **(A)** Satellite image of some collection points in the Amazon rainforest; **(B)** *Igapó* forest landscape at Ariau river, Amazonas, Amazon rainforest. **(C)** Satellite image of some collection points in the Atlantic forest; **(D)** *Restinga* forest landscape at Iguape, São Paulo, Atlantic forest.

Plants were collected from the Amazon (**Figures 1A, B**) and Atlantic forests (**Figures 1C, D**), two major Brazilian biomes that are considered hotspots in terms of their biodiversity and conservation (**Figure 1**). Plants were collected from areas under the jurisdiction of the Brazilian Institute of Environment and Renewable Natural Resources *(Instituto Brasileiro do Meio Ambiente e dos Recursos Renováveis*—IBAMA)—license numbers 12A/08 and 14879. Vouchers from each collected species were deposited at the UNIP-Herbarium (UNIP), where botanical identification was conducted. The plant organs that were used to obtain the extracts were collected according to the mass availability. The crude plant material to be extracted was cleaned by removing insects, parts of different plants, earth, sand, and other strange material. When cleaned, the crude plant material was placed in an air-circulating incubator (Fanem, Diadema, SP, Brazil) at 40°C until dry. After that, the plant material was ground using a hammer mill (Holmes, Danville, Illinois, USA) and a voucher containing the plant organs that had been ground was obtained. The ground material was packed in plastic bags, then sealed and identified with a label specifying the material’s ID collection number, collect number, voucher number, family, genus, species, and plant part, and kept in a cold chamber at −20°C until being subjected to extraction.

### 2.2 Plant extract and preparation

Dry and ground plant material was subjected to an extraction procedure that consisted of two macerations, each one lasting for 24 h. The first 24-hour maceration was carried out with a mixture of dichloromethane and methanol (at a 1:1 ratio). This was followed by a 24-hour maceration with pure distilled water. The solvents used in the first maceration were removed using a rotary evaporator (Buchi, Flawil, Switzerland), and the water was removed by lyophilization of the aqueous extract. The dry crude organic and aqueous extracts were kept in amber glass containers, with the organic extract containers being labelled with odd numbers and the aqueous extract containers being labelled with even numbers. The above procedure was used on all collected plant parts to obtain the 2,280 extracts used in the present study.

The organic extracts were diluted with dimethylsulfoxide 50% in water (DMSO50), and the aqueous extracts were diluted with water to a standard concentration of 100 mg/mL, from which lower dilutions were obtained. DMSO and DMSO50 were tested against all the microorganisms and showed no activity. Either chlorhexidine gluconate or amphotericin B (both at a concentration of 1%) were used as standard antimicrobials, depending on the microorganism being tested.

### 2.3 Susceptibility tests

The susceptibility tests conducted in the present study, disk diffusion assay (DDA) and microdilution broth assay (MBA), were developed based on Clinical & Laboratory Standards Institute—CLSI guidelines (CLSI, 2006a; CLSI, 2006b; CLSI, 2008a; CLSI, 2008b), with adaptations. DDA was used in the large-scale screening and to evaluate the inhibition zone diameter and MBA was used to obtain the minimum bactericidal concentration for each active extract. Figures related to the technique are shown in the **Supplementary Material**, **Figures S2**, **S3**.

#### 2.3.1 Microorganisms

The following microorganisms were used: *S. aureus* ATCC^®^29213™, *S. epidermidis* ATCC^®^12228™, *E. faecalis* ATCC^®^29212™, *S. mutans* ATCC^®^25175™, *S. sanguinis* ATCC^®^10556™, *L. monocytogenes* ATCC^®^7644™, *E. coli* ATCC^®^25922™, *P. aeruginosa* ATCC^®^27853™, *C. albicans* ATCC^®^10231™, *M. pachydermatis*, and *M. furfur*. The microorganisms were obtained from Oxoid^®^ Culti-Loops^®^, ThermoFisher Sci. *Streptococcus* spp. were obtained from KwikStiks^®^ MicroBiologics. *M. pachydermatis* CBS-1696 and *M. furfur* CBS-1878 were acquired from Fungal Biodiversity Center (CBS)—Fungal Strains. Microorganisms were first manipulated in accordance with the Oxoid^®^ manual procedures and rigorously tested in the third or fourth passage.

#### 2.3.2 Culture medium, microorganism growth conditions, and inoculum concentrations

For the culture media, Mueller–Hinton agar (MHA) and broth (MHB) were used for testing *S. aureus*, *E. faecalis*, *E. coli*, and *P. aeruginosa.* Brain–heart infusion agar (BHIA) and broth (BHIB) were used for the culturing of *Streptococcus* spp. Sabouraud dextrose agar (SDA) and broth (SDB) were used for the culturing of *C. albicans* and *Malassezia* spp. Oxford *Listeria* agar (OLA) and MHB were used for the culturing of *L. monocytogenes*.

*S. aureus*, *S. epidermidis*, *E. faecalis*, *E. coli*, and *P. aeruginosa* were incubated at 36°C for 24 hours. *Streptococcus* spp. were incubated at 36°C for 48 hours*. C. albicans* was incubated at 36°C for 48 hours. *Malassezia* spp. were incubated at 32°C for 48 hours. *L. monocytogenes* was incubated at 36°C for 24 hours.

The bacteria or yeast inoculum was prepared in a sterile saline solution as a suspension. Inoculum microorganisms were obtained from fresh colonies prepared in agar medium. Briefly, the bacteria/yeast concentration in the suspension was determined using the serial dilution technique, which consists of diluting the initial inoculum by carrying out a 1:10-fold serial dilution with saline solution (Castilho et al., 2013; da Silva et al., 2014; Martins et al., 2019a). Each of the dilutions was sampled and seeded in Petri dishes containing the appropriate agar medium. The plates were incubated for a sufficient length of time, and the colony-forming units of microorganisms were counted and used to establish the exact inoculum concentration, which is specific to each microorganism. The inoculum concentrations of saline solution or broth medium used in the agar disk diffusion or microdilution broth susceptibility tests, respectively, were as follows: the *S. aureus* and *S. epidermidis* inoculums were at 1.5 × 10^8^ CFU/mL; the *E. faecalis* inoculum was at 1.5 × 10^7^ CFU/mL; the *E. coli* inoculum was at 1.5 × 10^7^ CFU/mL; the *P. aeruginosa* inoculum was at 1.5 × 10^8^ CFU/mL; the *S. mutans* inoculum was at 1.5 × 10^7^ CFU/mL; the *S. sanguinis* inoculum was at 1.5 × 10^7^ CFU/mL; the *C. albicans* inoculum was at 1.5 × 10^5^ CFU/mL; the *M. pachydermatis* inoculum was at 1.5 × 10^6^ CFU/mL; the *M. furfur* inoculum was at 1.5 × 10^5^ CFU/mL; and the *L. monocytogenes* inoculum at was 1.5 × 10^8^ CFU/mL.

#### 2.3.3 Disk diffusion assay

The test was conducted with a single microorganism at a time (CLSI, 2006a; CLSI, 2008a; CLSI, 2008b). Ten mL of a saline solution prepared using the suitable inoculum was used for surface inoculation of the microorganism on 90-mm Petri dishes prepared with 15 mL of the suitable agar medium. Subsequently, 6-mm sterile paper disks were distributed over the inoculated agar medium, and 10 μL of the treatment was added to each paper disk. The plates were set on the benchtop for 2 minutes before being incubated under the appropriate conditions. After being incubated for the same period as that previously described for each microorganism, the Petri dishes were removed from the incubator and the formation of the inhibition zone was observed.

##### 2.3.3.1 DDA in the large-scale screening

DDA was used for largescale screening, and involved each plant being tested once at the same concentration. To avoid cross-contamination or misinterpretation of the results, each paper disk to which the treatment was added was identified by the treatment ID being written on the Petri-dish lid, and by our drawing a mark from the lid to the body of the Petri dish, so that the disk and treatment ID coincided. Subsequently, 10 μL of each treatment was added to the corresponding disk. Each 90-mm Petri dish could hold up to 19 disks. Larger Petri dishes could have been used, although there would not have been much gain in terms of costs, considering the larger amount of medium needed. The Petri dishes were set for two minutes before they were placed in the incubator. After being incubated for the same period as that previously described for each microorganism, the Petri dishes were removed from the incubator, and the results read. The formation of any inhibition zone around a disk was considered a positive result.

##### 2.3.3.2 DDA to obtain the inhibition zone diameter

DDA was also used to obtain the inhibition zone diameter, or IZD. Briefly, 90-mm Petri dishes were prepared and identified in accordance with the method described previously, and underwent in-surface inoculation with the appropriate inoculum. Each dish held no more than six units of 6-mm paper disks arranged in the appropriate distribution. Each treatment was assayed in triplicate at the same concentration that had been used in the screening assay. The Petri dishes were set for 2 minutes, so that the disks did not lose from the medium surface before they were placed in the incubator. After being incubated for the same period as that previously described for each microorganism, the Petri dishes were removed from the incubator, and the results were read. IZDs were obtained using a caliper rule, with diameters given in mm. Finally, six measurements were obtained for each active extract; these were used in the statistical calculations.

#### 2.3.4 Microdilution broth assay

The microdilution broth assay (MBA) was conducted with the active plant extracts to obtain the minimum bactericidal concentrations, or MBCs (CLSI, 2006a). The assays were conducted in sterile U-shape 96-well microplates. Plates with or without lids could be used according to the budget of the lab in question. If plates do not have covers, a cover made with Parafilm™ or a similar product could be improvised. Each microplate can test up to seven plant extracts at six different concentrations, all in duplicate. Positive and negative bacteria growth controls in each microplate were added. A volume of 190 μL of inoculated broth medium was added to each corresponding well. After that, 10 μL of the treatment at the appropriate concentration was added to the corresponding well. The final concentrations of the samples being analyzed corresponded to a 1:20 ratio based on the initial dilution. The microplates were closed with Parafilm^®^ and incubated at the adequate temperature and time as stipulated for each microorganism. Subsequently, the controls were visually evaluated to check for contamination then assessed for the presence or absence of turbidity in each well. As plant extract may sometimes not adequately dilute in the broth medium, leading to misinterpretations, it is mandatory to conduct a subculture to check for bacteria growth in all the wells. The following parameters were adopted: “X” = microorganism growth deposited in the bottom of the well, no inhibition; “C” = turbidity formation in a “cloudy” pattern, flocculation without deposit in the bottom of the well; “++” = numerous flocculation, absence of turbidity; “+” = few flocculations, absence of turbidity; and “L” = clean medium, probable microorganism growth inhibition—subculture required. The “L” results were subcultured in a specific agar medium according to the microorganism’s growth conditions.

Petri dishes prepared with 15 mL of the corresponding agar medium were used in the subculture. A 2-μL volume of medium from each well was collected and seeded in the Petri dish in areas designated to each sample. The Petri dishes containing the subculture were taken to the incubator and after the appropriate incubation time previously stipulated had passed, the dishes were removed from the incubator and read. Results from the MBA indicated the minimum inhibitory concentration or minimum bactericidal concentration. If there was microorganism growth in the subculture, the treatment was classified as “L+”, and if there was no microorganism growth, the treatment was classified as “L–”. MICs (minimum inhibitory concentrations) were obtained if the treatment was classified as “L+,” and MBCs (minimum bactericidal concentrations) were obtained if the treatment was classified as “L–”. The visual evaluation of the colored plant extracts is complemented with a subculture independently of the “L” classification.

### 2.4 Bioautography

#### 2.4.1 Preparing the thin-layer chromatograms as support for bioautography

Thin-layer chromatography (TLC) silica gel G254 deposited over 5.0 × 5.0 cm aluminum support plates was used in the bioautography assays. As the natures of organic and aqueous plant extracts are different, as is the composition of each extract itself, the mobile phase containing a mixture of ethyl acetate, methanol, and water (at a 100:35:10 ratio) (Wagner and Bladt, 1996) was used in the bioautography assay. A 250-mL glass beaker with a glass lid was used as the chamber for the elution of the TLC plaques. A volume of 10 mL of the mobile phase was added to each beaker.

#### 2.4.2 Drop diffusion in bioautography

The plant extracts tested were of the same concentration as those that were used in the DDA. The crude extracts were applied in volumes of 2.0 μL, 5.0 μL, and 10.0 μL, in triplicates, in the shape of a triangle, with one TLC plate for each volume per extract. The solvents used to dilute the plant extracts were removed by air-drying. The TLC plates were put in sterile 90-mm Petri dishes under sterile conditions and 15 mL of inoculated agar medium was poured onto the TLC plates. The Petri dishes were placed in an incubator, where they remained for the necessary length of time at a suitable temperature. After the corresponding period of incubation, inhibition zones were observed over the extract spots. For better visualization, 0.5 mL of the cell viability dye MTT [3-(4,5-dimethylthiazol-2-yl)-2,5-diphenyltetrazolium bromide], a yellow tetrazolium salt, was added to the dish at a concentration of 0.5 mg/mL. The dishes were incubated for 4 hours so that MTT could react with the mitochondria of viable microorganisms and form the purple formazan salt. The inhibition zone diameters, IZDs, which were taken as the non-colored area of the medium over a specific spot in the TLC chromatogram with active compounds, were measured with a caliper rule in mm. A DiB assay was developed to analyze the extract behavior in diffusing from the paper disk used in the DDA and from the TLC stationary phase composed of silica gel. This method was employed to select which extracts could be fractionated by preparative TLC from those to be subjected to any other fractionation technique. The ratio “r” between DDA and DiB IZD (r = DDA/DiB) was obtained for each extract analyzed in the same concentration of 100 mg/mL, at an equal volume of 10 μL. It is expected that “r” tends to one when diffusion occurs equally in the paper disk and the TLC silica gel, r >1 when DDA IZD > DiB IZD, and r <1 when DDA IZD < DiB IZD.

#### 2.4.3 Unidimensional and bidimensional bioautography

Each plant extract was subjected to unidimensional and bidimensional TLC. In the unidimensional TLC, 10 μL of each extract was applied on the plate along a 2-cm line, with two extracts being applied to each plate. The elution was made in a 250-mL beaker chamber covered with a glass lid and ended up to 4 cm from the application point. The chromatogram was placed in the chamber so the mobile phase solvents were evaporated entirely and did not influence the biological response of the bioautography.

The bidimensional bioautography was conducted using the same method as the unidimensional bioautography, except for the elution procedure. Although two plant extracts were analyzed in a single TLC plate in the unidimensional bioautography, only one extract was studied in the bidimensional bioautography. Briefly, 10 μL of the plant extract was applied as a spot in the right inferior area of the TLC plate. The TLC plate was eluted in a 250-mL beaker chamber with a glass lid up to the 4-cm front-line. The chromatogram was left to air-dry. Subsequently, the same chromatogram was rotated 90° counterclockwise before being put in the beaker chamber to a new elution up to the front line of 4 cm. After the elution, the chromatograms were placed in the chamber to allow the solvents of the mobile phase to fully evaporate, so as not to influence the biological response.

The chromatograms were placed in sterile 90-mm Petri dishes and 15 mL of the microbial suspensions was inoculated in a sterile agar culture medium at the desired concentration. The Petri dishes were placed in an incubator, where they remained for the necessary length of time at a suitable temperature. After the corresponding incubation period, inhibition zones were observed over the extract spots. For better visualization, 0.5 mL of the cell viability dye MTT was added to the dishes, as reported earlier in section 2.4.2.

### 2.5 *Artemia salina* toxicity assay

The *Artemia* toxicity assay (Meyer et al., 1982) was first developed to evaluate the acute toxic effects of a chemical compound. Responses are given a EC50, corresponding to the chemical compound concentration capable of eliminating 50% of the nauplii after 24 hours and 48 hours. 

A volume of 500 mL of marine water was added to a rectangular plastic container with a capacity of 1.0 L. The reconstituted marine water was made by adding 15 g of aquaculture sea salt to 500 mL of Milli-Q water. The container was divided into two parts, with a plastic separator that allowed communication between these two parts and the passage of the nauplii after the cysts hatch. A total of 3 g of *Artemia* cysts were placed in the smaller part of the container, which remained covered with foil so that it was protected from light. The cysts remained in the aquarium until they hatched approximately 48 hours later. A lamp was placed to illuminate the larger part of the aquarium. After hatching, the nauplii born on the dark side of the aquarium passed to the light side, attracted by the light. On the day of hatching, 10 nauplii were transferred with Pasteur pipette to each well of 24-well plates. After transferring the nauplii, each well was topped up with seawater to a final volume of 3 mL. To keep the nauplii fed, a drop of biological yeast diluted in water was added to each well, with a final concentration of 1.67 μg in 3 mL. The 24-well microplate lids were not used, which allowed constant air exchange in the wells. The nauplii were kept under these conditions for 24 hours.

The toxicity experiments were carried out with the 24-hour nauplii in 24-well microplates. Two experiments were carried out: the first was conducted with the extracts being tested at a single dose of 1.0 mg/mL, which served as a toxicity screening to separate the toxic from the non-toxic extracts; and the second experiment was carried out with the toxic extracts, and three more doses of extracts at concentrations of 0.5 mg/mL, 0.1 mg/mL, and 0.03 mg/mL were used. All experiments were carried out in triplicate. After 24 hours and 48 hours of nauplii–extract contact, the results were analyzed by counting the remaining living nauplii. For the calculations of the 50% effective concentration (EC50), the numbers of dead nauplii were considered according to each dose adopted. Thus, the dose–effect curve and the values corresponding to the EC50 for each treatment were obtained. Figures related to the *Artemia salina* toxicity assay can be are presented in **Figure S5** in the **Supplementary Material**.

### 2.6 Extract ranking

The extracts selected by the large-scale DDA were subjected to tests to obtain IZDs based on DDA and in DiB, tests to obtain CBMs based on MBA, bioautography, and the *Artemia* toxicity assay. All the assays are relevant to the classification of the extracts according to their potential as antimicrobials. Nonetheless, each extract resulted in a different classification of extract potentiality. To correct that inconvenience, we proposed a method based on establishing scores for each assay conducted on an individual extract, which can be seen in **Table 1**. The scores are summed and an adequate classification is obtained for the extracts. To exemplify the methodology, only the extracts subjected to the DDA, DiB, and MBA conducted with *S. aureus, S. epidermidis, S. mutans, C. albicans*, and *L. monocytogenes*, and to the *Artemia* toxicity assay, were considered in the ranking.

Table 1Scores for each assay used to establish the plant extract ranking analysis.

**Table:** 

Score	Diffusion assays—DDA and DiB
1	Significance *p* < 0.0001 ****
2	Significance *p* < 0.0001 ***
3	Significance *p* < 0.01 **
4	Significance *p* < 0.05 *
5	*p* > 0.9991 and mean rank difference <100
6	*p* > 0.201 and mean rank difference < 200; if mean rank differences are in specific range, consider the p-value
7	0.20 >*p* > 0.05

**Table:** 

Score	Microdilution broth assay performed with previously established concentrations, in mg/mL
5	0.039 and 0.156
4	0.25 and 0.313
3	0.5 and 0.625
2	1.25, 2.5, and 5.0
1	10, 12.5, and 15

**Table:** 

Score	*Artemia* immobility toxicity assay, in mg/mL
1	0 to 0.200
2	0.200 to 2.00
3	2.01 to 5.0
4	5,01 to 10.0
5	10.01 to 40.0
6	40.01 to 100.00
7	100.01 and above

DDA and DiB scores were based on Tukey’s mean or Dunn’s median comparison tests carried out with the inhibition zone diameters’ median and either 10 µL chlorhexidine gluconate 1% or amphotericin B 50 mg/mL, the reference substances. The statistical analyses were conducted in accordance with the method described in section 2.8.

MBA scores were attributed to the minimum bactericidal concentrations obtained based on six 1/2-fold dilutions starting from 10 mg/mL. Scores from 1 to 5 were given (where 1 indicated the highest MBC, and 5 the lowest).

The scores from the *Artemia* toxicity assay were related to the EC50 values.

### 2.7 Experimental design

**Figure 2:**
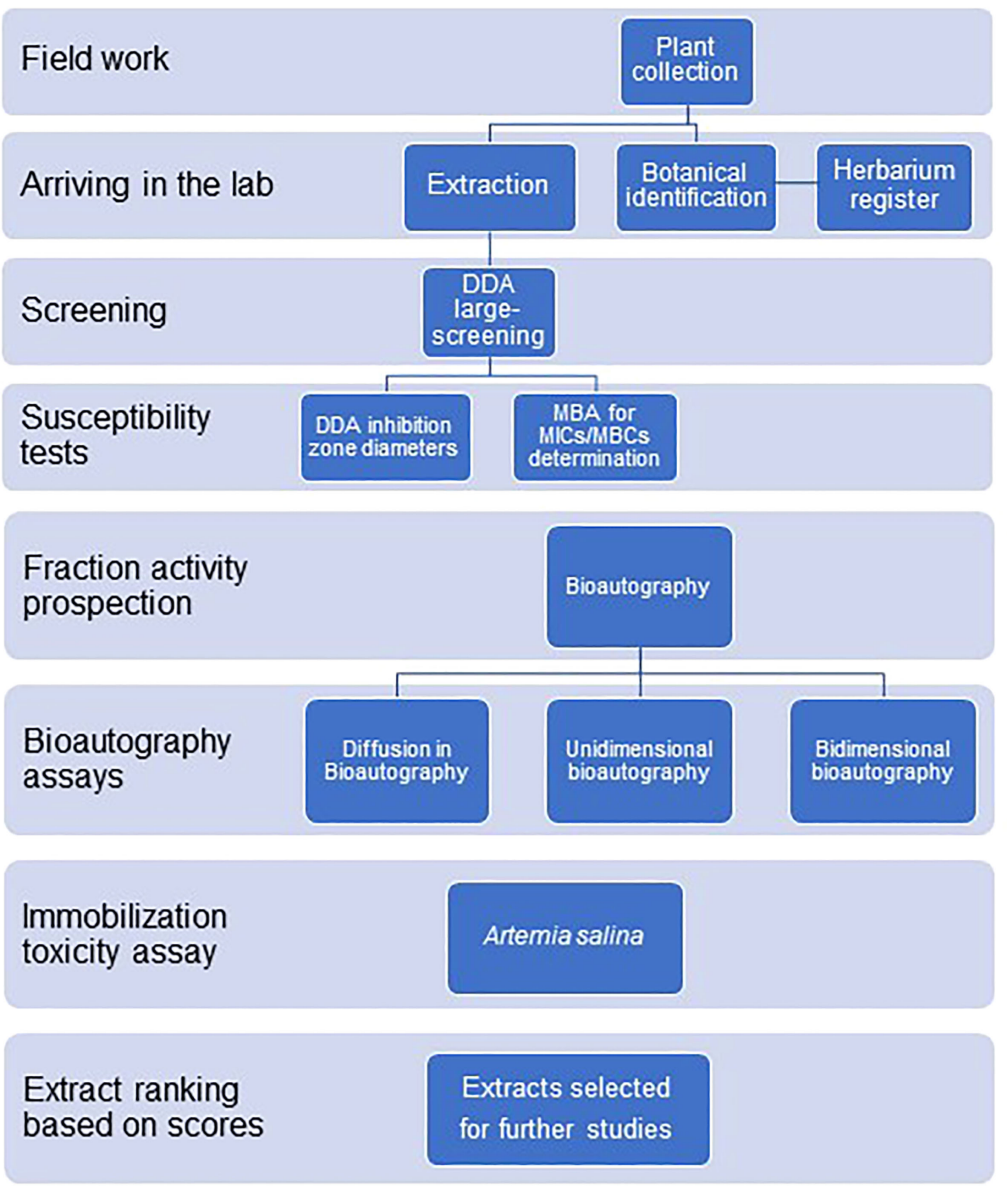
Experimental design for the antimicrobial large-scale screening of plant extracts from the Amazon rainforest and the Atlantic forest.

**Figure 2** shows the experimental design used in the large-scale screening procedures.

### 2.8 Statistics

Principal component analysis (PCA) was carried out using the IZDs obtained both from DDA and DiB and also with the minimum bactericidal concentrations obtained from MBA (MultiVariate Statistical Package 3.22 Kovach Computing Service 1985–2013).

The extract’s antimicrobial effectiveness comparison used DDA and DiB IZD data. All measurements were obtained from independent experiments. Data were subjected to the Kolmogorov–Smirnoff normality test. The identification of outliers was carried out with the ROUT-test (Q = 5%). After that, Kruskal–Wallis and Dunn’s median comparison tests or one-way ANOVA and Tukey’s mean post tests were carried out. The level of significance was set as α < 0.05. In addition, data were used to obtain the ratio between DDA and DiB IZDs (GraphPad Prism 7.05, GraphPad Software Inc., 2018).

The Finney's test for probit analysis was used to calculate the 50% effective concentrations (EC50s) in the *Artemia* toxicity assay.

## 3 Results

In the present study, 17 aqueous and 137 organic plant extracts (154 in total) out of 2,280 extracts, or 6.75%, showed activity against one or more microorganisms from a selection based on a large-scale screening in disk diffusion assay. The extracts were obtained from 139 different plant species that belonged to 91 genera and 44 families, such as the Acanthaceae, Annonaceae, Apocynaceae, Aquifoliaceae, Araceae, Bignoniaceae, Boraginaceae, Burseraceae, Caryocaraceae, Celastraceae, Chrysobalanaceae, Clusiaceae (including Hypericaceae and Calophyllaceae), Combretaceae, Connaraceae, Convolvulaceae, Dioscoreaceae, Ebenaceae, Elaeocarpaceae, Fabaceae (including Caesalpinioidae, Mimosoidae, and Faboidae), Gentianaceae, Gnetaceae, Hippocrateaceae, Lauraceae, Lecythidaceae, Linaceae, Malpighiaceae, Moraceae, Myristicaceae, Myrsinaceae, Myrtaceae, Olacaceae, Orchidaceae, Piperaceae, Polygonaceae, Proteaceae, Rhabdodendraceae, Rhizophoraceae, Rubiaceae, Rutaceae, Salicaceae (formerly Flacourtiaceae), Sapindaceae, Sapotaceae, Simaroubaceae, Solanaceae, Vitaceae, and Vochysiaceae families.

### 3.1 Large-scale screening of antimicrobial plant extracts based on DDA

**Table 2** reports the number of extracts subjected to the DDA screening tests, the number and percentage yield of active extracts, and the corresponding number of susceptible microorganisms to each of the 154 active extracts. The DDA susceptibility test was used in the screening because it is a classical test recommended by the CLSI to verify which antibiotics can suitably be used against a specific pathogenic microorganism, and that favors reproducibility. Of the 154 active extracts, 73% were active against only one microorganism, 19% were active against two microorganisms, 5% against three microorganisms, and 1% against four, five, or six microorganisms.

**Table 2:** Number of plant extracts screened, and number and percentage yield of active plant extracts against each microorganism.

Microorganism	Number of plant extracts screened	Percentage yield of active plant extracts	Percentage yield	K–S	p-value
*Staphylococcus aureus (* Pinto, 2020)	2,236	73	3.26%	0.1509	0.0006
*Staphylococcus epidermidis (* Belo, 2022)	2,240	20	0.89%	0.2016	0.0323
*Streptococcus mutans (* Silva, 2009)	2,000	18	0.9%	0.1543	>0.1000
*Streptococcus mutans (* Bento, 2020)	2,240	7	0.31%	0.2064	>0.1000
*Streptococcus sanguinis (* Silva, 2009)	2,000	37	1.85%	0.1596	0.0182
*Enterococcus faecalis (* Castilho, 2009)	2,000	25	1.25%	0.1451	>0.1000
*Listeria monocytogenes (* Ceruso et al., 2020)	2,280	12	0.53%	0.1833	>0.1000
*Escherichia coli (* Camargo and Suffredini, 2014; Camargo et al., 2020)	1,791	4	0.22%	*n* too small	
*Pseudomonas aeruginosa*	1,300	0	0%	*n* too small	
*Candida albicans (* Rudiger, 2021)	2,240	6	0.27%	0.2917	>0.1000
*Malassezia pachydermatis (* Silva, 2018)	2,240	11	0.49%	0.2809	0.0154
*Malassezia furfur (* Silva, 2018)	2,240	7	0.31%	0.2477	>0.1000

**Figure 3:**
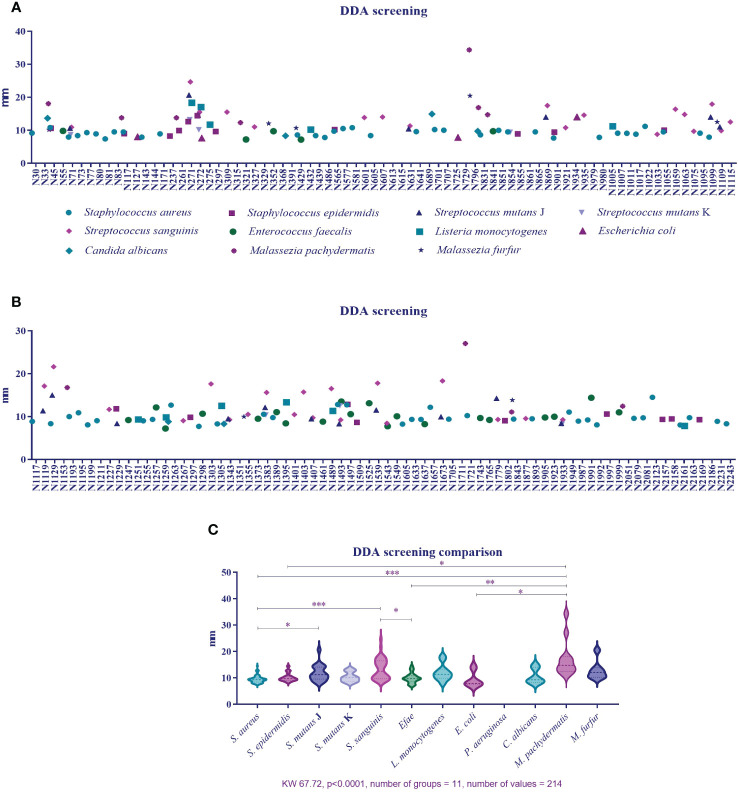
**(A)** Percentage of active plant extracts against a number of susceptible microorganisms obtained from disk diffusion assays in the large-scale screening. **(B)** Inhibition zone diameter means obtained from the plant extracts’ activity against 11 microorganisms. **(C)**. Comparison of inhibition zone diameters as a response to plant extract activity among microorganisms.

**Figures 3A, B** qualitatively show the 154 active plant extracts selected from the large-scale screening conducted with the eleven microorganisms. **Figure 3C** compares the plant extracts antimicrobial activity potential among the bacteria. The Kolgomorov–Smirnoff normality test showed that data from *S. aureus, S. epidermidis, S. sanguinis*, and *M. pachydermatis* did not fit a normal distribution, even after removing outliers, so the Kruskal–Wallis test was conducted and resulted in KW 67.72 (*p* < 0.0001, number of groups = 11, and number of values = 214). Extracts produced less sensitivity in *S. aureus* than in *S. mutans* (*p* = 0.0229), *S. sanguinis* (*p* < 0.0001), and *M. pachydermatis* (*p* < 0.0001). *S. epidermidis* was less susceptible to the extracts than *M. pachydermatis* (*p* = 0.0115). At the same time, *S. sanguinis* was more affected by the extracts than *E. faecalis* (*p* = 0.0207). *E. faecalis* and *E. coli* were less affected by the extracts than *M. pachydermatis* (*p* = 0.0014 and *p* = 0.0138, respectively).

### 3.2 Determination of the inhibition zone diameter of antimicrobial plant extracts based on DDA

**Figure 4:**
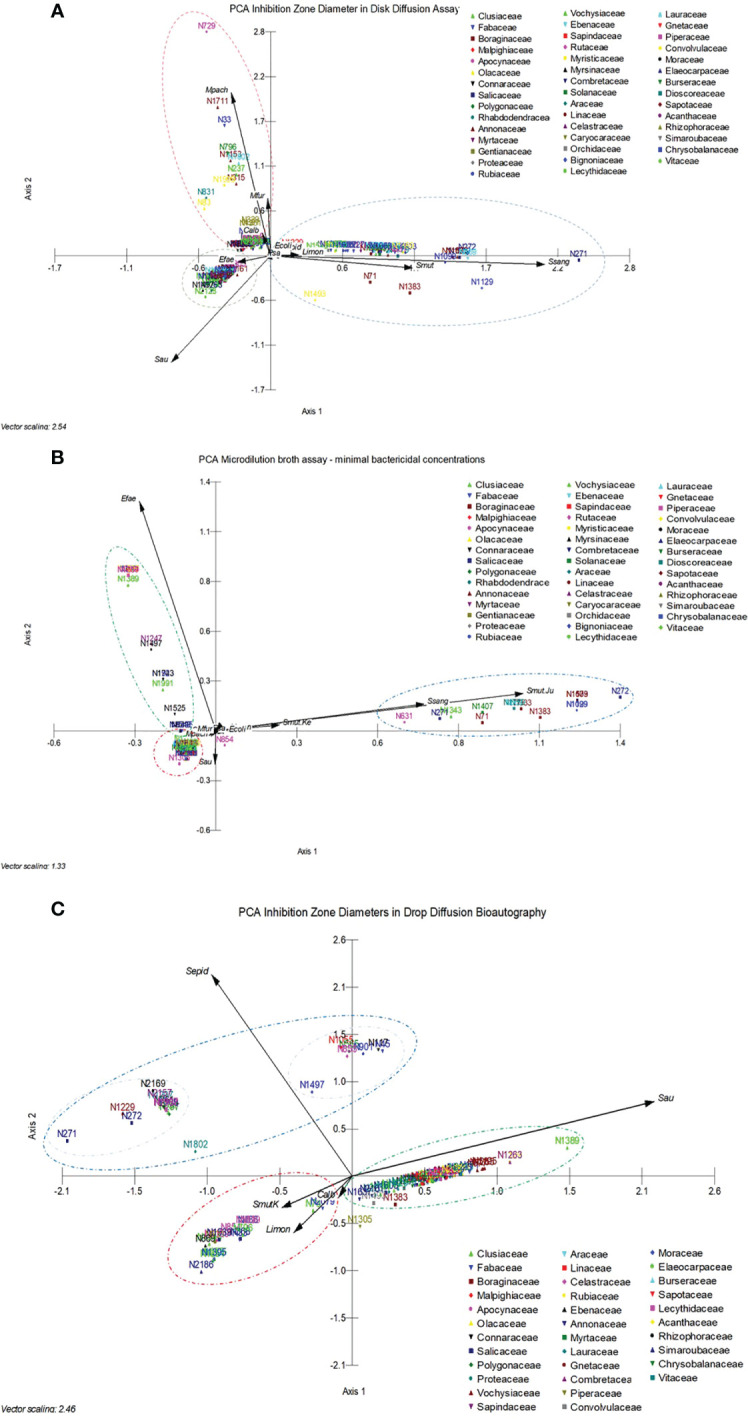
**(A)** Principal component analysis carried out with 11 different microorganisms, considered the variables, and the 154 active extracts, considered the cases, analyzed in the disk diffusion assay to obtain the inhibition zone diameters. **(B)** Principal component analysis carried out with 11 different microorganisms and a microorganism that was tested twice, considered the variables, and the 154 active extracts, considered the cases, analyzed in the microdilution broth assay to obtain the minimum bactericidal concentrations. **(C)** Principal component analysis carried out with five different microorganisms, considered the variables, and 96 active extracts, considered the cases. Data refer to the minimum bactericidal concentrations, obtained from the microdilution broth assays.

The selected active plant extracts were subjected again to DDA to obtain IZDs. We chose to use the DDA susceptibility test for the determination of IZDs because it had previously been used in the screening procedure and as a strategy to confirm the antimicrobial activity of the selected extract, and because we aimed to make this process reproducible. Six measurements were obtained from three paper disks for each extract. A PCA was conducted and **Figure 4A** shows the results, where 11 different microorganisms were considered the variables and the 154 active extracts were considered the cases. A cumulative percentage of 52.250% was obtained on axis 2, and 74.241% on axis 4. A gradient and two clusters along axis 1 can be observed. The first and smallest cluster was directly influenced by *E. faecalis* and *S. aureus*, whereas *S. sanguinis, S. mutans, L. monocytogenes*, and *S. epidermidis* influenced the second cluster. A third cluster can be observed along axis 2 and is influenced by *M. pachydermatis, M. furfur*, and *C. albicans*. Interestingly, clusters were most influenced by Gram-positive bacteria and yeasts, and *E. coli* and *P. aeruginosa* had the least clustering influence.

### 3.3 Minimum bactericidal concentration of the antimicrobial plant extracts

The selected active plant extracts were also subjected to MBA to obtain MBCs. The MBA susceptibility test was used in the screening because it is a classical test that is recommended by the CLSI for the determination of minimum inhibitory concentrations, which are specific to a given substance or, in our case, to specific plant extracts, which are complex mixtures of substances. The MBA test also supports reproducibility once it is routinely done in microbiology labs. A PCA was carried out, and **Figure 4B** shows the results that were obtained from 11 different microorganisms, which were considered the variables, and the 154 active extracts, which were considered the cases. A cumulative percentage of 70.677% was obtained on axis 2, and a cumulative percentage of 79.528% was obtained on axis 3. A gradient along axis 1 and two clusters can be observed. The first and small cluster is directly influenced by *S. aureus*, *M. pachydermatis*, and *M. furfur*, whereas *S. sanguinis, S. mutans*, and *L. monocytogenes* influence the second cluster. The third cluster can be observed along axis 2 and is influenced by *E. faecalis*. In the analysis, the *E. coli* and *P. aeruginosa* results showed that they had the least clustering effect.

### 3.4 Determination of the inhibition zone diameter of antimicrobial plant extracts based on DiB

Drop diffusion in bioautography was conducted with five microorganisms, considered the variables, and with 101 cases in the PCA, shown in **Figure 4C**, to obtain the IZDs. A cumulative percentage of 75.653% was obtained in axis 2, and three clusters can be observed, influenced by *S. epidermidis, L. monocytogenes*, and *S. aureus*. The cluster influenced by *S. epidermidis* showed two subgroups influenced by the vectors corresponding to *S. aureus, S. mutans, L. monocytogenes*, and *C. albicans*.

#### 3.4.1 Comparison between DDA and DiB inhibition zone diameters

**Figure 5:**
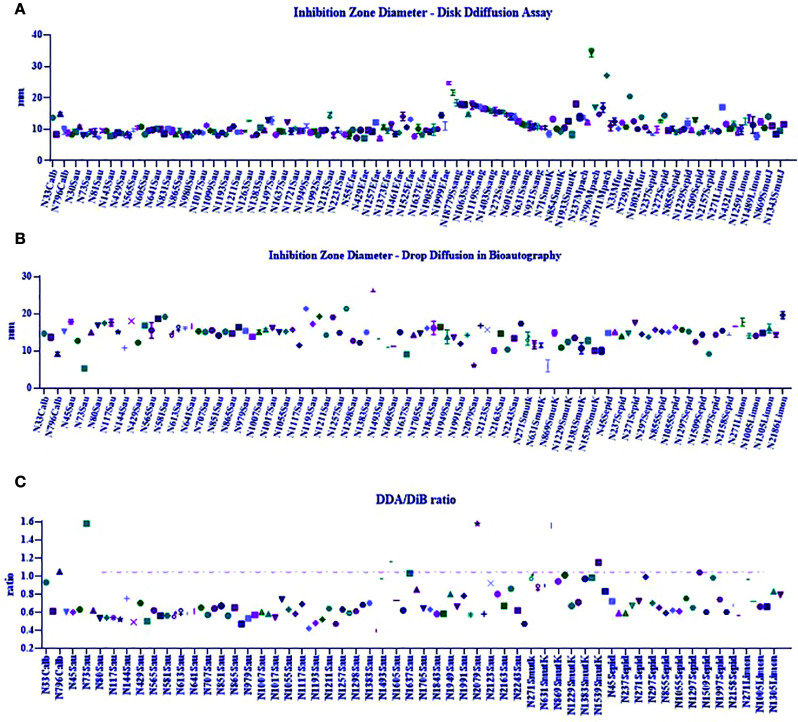
**(A)** Non-parametric analyses of variance performed to compare antimicrobial efficacy of plant extracts related to the inhibition zone diameters (IZDs) obtained from disk diffusion assay (DDA); **(B)** Non-parametric analyses of variance performed to compare antimicrobial efficacy of plant extracts related to the inhibition zone diameters (IZDs) obtained from the drop diffusion in bioautography assay (DiB). **(C)** Ratio between DDA and DiB IZDs.

A non-parametric analysis of variance was conducted to compare the IZDs obtained from DDA (K-S = 1021, 191 groups, number of values = 1142, *p* < 0.0001; **Figure 5A**). The statistical differences between medians (Dunn’s medians comparison post test) are shown in the **Supplementary Material**, Table A. A graph depicted in **Figure 5B** shows the results obtained from the non-parametric analysis made to compare the medians of IZDs obtained using DiB (K-S = 372.4, 111 groups, number of values = 390, *p* < 0.0001). The ratios between DDA and DiB IZDs were determined for each active extract and are shown in **Figure 5C**.

### 3.5 Verification of the qualitative presence of inhibition zones in UB and BB spots

All extracts that showed activity in DiB were subjected to UB and BB. The analyses resulted in a positive or negative answer based on whether or not a spot appeared over a fraction of the eluted extract, meaning that the substances in the specific area had diffused to the inoculated agar and inhibited microorganism growth. Of the 104 extracts that were analyzed using DiB, antimicrobial activity was confirmed in only 85 extracts (81.73%) by UB and BB.

### 3.6 *Artemia* toxicity of the active plant extracts

**Figure 6:**
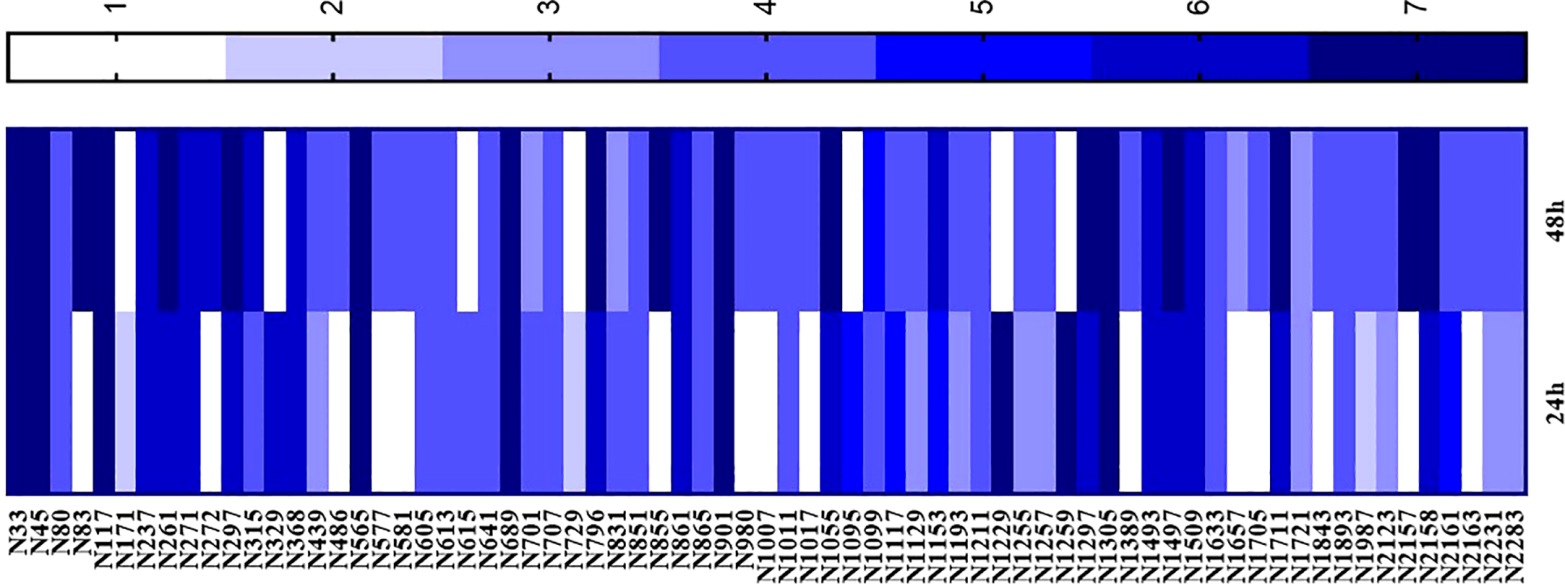
*Artemia* toxicity assay performed with plant extracts with activity against *Staphylococcus aureus, S. epidermidis, Streptococcus mutans*, *Malassezia* spp., and *Candida albicans*. Data are given in mg/mL and EC50 was obtained after 24 hours and 48 hours of extract exposure.

Eighty-four plant extracts with activity against *S. aureus, S. mutans, C. albicans*, and *Malassezia* spp. were tested in the *Artemia* toxicity assay over two rounds. In the first round, the extracts were tested at a concentration of 1 mg/mL, and those that were toxic for more than five nauplii were tested at lower concentrations in the second round. The extracts that were not toxic for at least five nauplii were considered “non-toxic”. Of the 84 extracts, 71 showed toxicity at 1 mg/mL and were subjected to a concentration × response test. EC50s were classified according to toxicity intensity. Scores from 0 to 7 were given, where 0 indicated the absence of toxicity and 7 indicated high toxicity. **Figure 6** shows the extracts that presented some degree of toxicity. The extracts that were classified as non-toxic were not included in the graph.

### 3.7 Extract ranking

**Figure 7:**
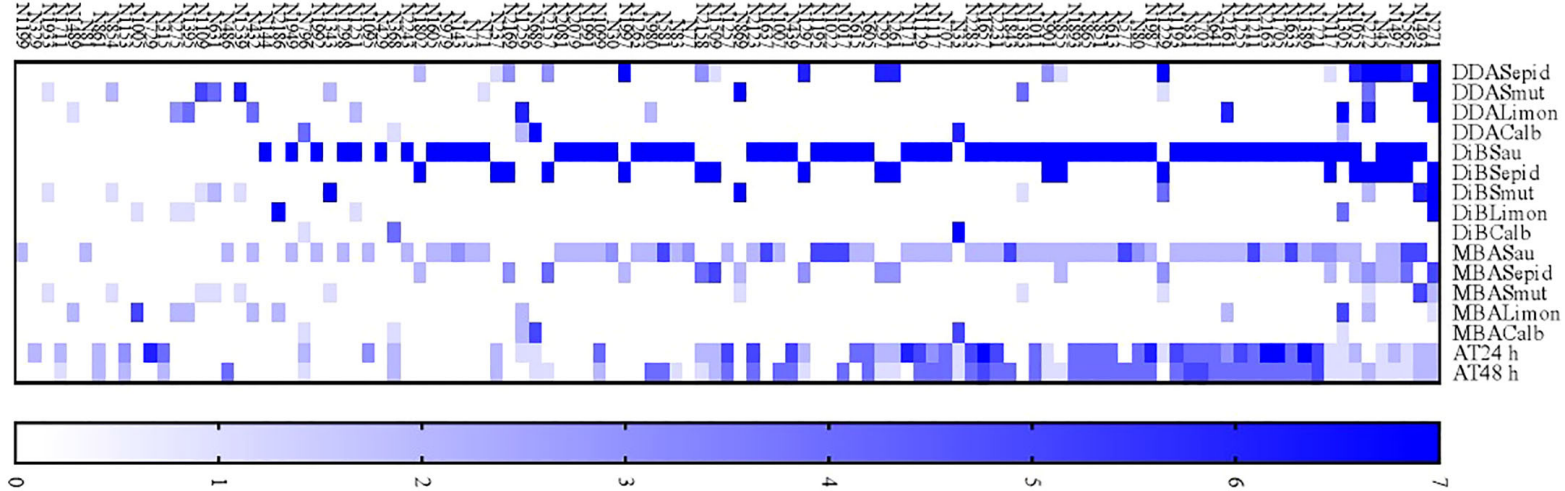
A proposed ranking of 111 plant extracts according to their antimicrobial activity efficacy and low toxicity.

According to the test results, 111 active extracts were used to test the proposition of a ranking based on scores. The ranking can be seen in **Figure 7**.

## 4 Discussion

Screening programs aiming to identify antimicrobials from natural settings must be accelerated due to the high rates of forest loss. Costs and access to remote places are barriers to accelerating the process of identifying active substances and collecting taxonomically known plants. The Amazon rainforest and the Atlantic forest are known for their rich biodiversity, as are other Brazilian biomes, such as the Cerrado, Pantanal, Caatinga, and Pampas, some of which contain land that is used for farming, which is the country’s primary source of wealth (Brazil, 2022). It is estimated that there are approximately 73,000 tree species in the world: 31,100 of them are in South America (Cazzolla Gatti et al., 2022); 16,000 in the Amazon rainforest (Steege et al., 2013); and approximately 4,000, mainly in the Amazon rainforest, are yet to be discovered (Cazzolla Gatti et al., 2022). Because the costs and logistics needed to support expeditions, professionals, lab infrastructure, and a constant consumable supply are high and demanding, there is a great need to optimize each step of the natural product-based research program for identifying active compounds, starting from plant collection.

The approach to plant collection is fundamental to establishing the overall strategy adopted for the fieldwork. Plant collection can be carried out based on information related to plants’ popular use, be supported by chemotaxonomic data, or be carried out at random. Usually, research groups prefer to study plants that have already undergone a selection process, aiming to search for molecules with specific pharmacological activities directed by traditional use. The efficacy of this process is evident in the list of natural products that are incorporated in medicine as tools to treat diseases, such as vinca alkaloids, scopolamine, atropine, morphine, codeine, rutin, and digoxin (Newman and Cragg, 2020).

Since the 1990s, plant collection has been consistently undertaken by our team. Fertile plant collections were randomly made. Special attention was given to species belonging to families known to include plants with traditional uses, such as Apocynaceae, Clusiaceae, Burseraceae, Lauraceae, Myrtaceae, Myristicaceae, Rubiaceae, and Solanaceae. The UNIP Herbarium and Extract Library is composed of plant samples from the Amazon igapó and *terra firme* forests, and Atlantic Forest *restinga*, or lowland ombrofilous forest. Plant organs were collected according to mass availability. The tree size hinders access to the canopy for the collection of “aerial organs”. All plant material was processed in the same way, so the procedure standardization allowed us to compare the biological results obtained for extract analyses.

Brazilian scientists have made many efforts to search the country’s immense biodiversity for biological activities. The antibacterial activity of 88 extracts obtained from Atlantic forest plants was assessed against *S. aureus* and *P. aeruginosa*; this showed that the most promising organic extract was that obtained from *Miconia latecrenata* (DC.) Naudin (Rodrigues et al., 2021). Extracts obtained from 26 plants from the southeast region of Brazil were tested against *Aeromonas hydrophila, Bacillus subtilis, P. aeruginosa*, and *S. aureus*, and the extracts obtained from *Lantana fucata* Lindl. (= *L*. *lilacina* Desf.) and *Phyllanthus tenellus* Roxb. showed the best antimicrobial activity (Oliveira et al., 2007). Sixty extracts obtained from a periodically flooded forest called Pantanal, located in the Central-West region in Brazil, were tested against *C. albicans* (Brighenti et al., 2017), *S. mutans*, and other oral-disease relevant bacteria (Brighenti et al., 2014), which resulted in the selection of active extracts from *Buchenavia tomentosa* Roxb. and *Croton doctoris* S.Moore. Twenty-three extracts obtained from plants from the Caatinga, which is a semiarid biome from northeast Brazil, were tested against the biofilm-forming *S. epidermidis* and *P. aeruginosa*; this resulted in the identification of active extracts obtained from *Apuleia leiocarpa* (Vogel) J.F.Macbr (Silva et al., 2015).

The interest in finding plant extracts that are active against human pathogens is well documented and has been pursued for decades. The interest in finding antimicrobial extracts that can target veterinarian pathogens has been growing recently. *Staphylococcus* spp. and *E. coli* isolated from cattle were the targets of a study conducted with 13 leaf extracts obtained from plants collected in the Cerrado, a biome that is characteristic of the central area of Brazil, which is the area where the majority of animal meat is produced for export. Extracts from *Schinopsis brasiliensis* Engl.*, Annona crassiflora* Mart., and *Caryocar brasiliense* Cambess were found to be the most active against *Staphylococcus* spp. and *E. coli* (Ribeiro I.C. de et al., 2018). Attempts to find active extracts against pathogenic bacteria in fish (i.e., *S. agalactiae, Flavobacterium columnare*, and *Aeromonas hydrophila)* were successful for 31 out of 46 extracts obtained from plants collected in the Southeast region, namely *Merremia tomentosa* (Choisy) Hallier f., *Myrcia neoclusiifloia* A.R.Lourenço & E.Lucas (= *Calyptranthes clusifolia* O.Berg), and *Myrcia splendens* (Sw.) Dc. (= *M. velutina* O.Berg) (Castro et al., 2008).

In the present study, our group reports on the efforts of a 15-year project to conduct a large-scale screening of 2,280 Brazilian plant extracts. We provide information on 154 active extracts obtained from 139 different plant species, belonging to 91 genera and 46 families, making this the largest academic screening project available in the literature up to the present day, and, to our knowledge, the largest ever such project to be reported in Brazilian territory, and one that will serve as a basis for future phytochemical projects.

When developing this project, we chose a simple, fast, easy-to-conduct, low-cost, reproducible methodology for use in the large-scale screening. The disk diffusion assay, DDA, which can be used for the screening of plant extracts, met these criteria. DDA (CLSI, 2006a; CLSI, 2008a; CLSI, 2008b) is a classical susceptibility assay that Kirby and Bauer described in 1966 to predict therapeutic conduct in the use of antibiotics (ANVISA, 2022) and developed earlier in the 1940s (Heatley, 1944). Although it was established many years ago, it remains a simple and reliable qualitative assay that meets the criteria for practicality needed of large-scale hand-made techniques. Some advantages of this assay are its ease of execution, its reproducibility, the low cost of the required reagents, the easy-to-interpret results it produces, that it is adaptable enough to be carried out with different microorganisms, and that it requires no special equipment, all of which makes it suitable for testing large amounts of extracts. As for the limitations of this method, specific microorganisms can grow only in a known medium, and under specific conditions of temperature, atmosphere, and humidity, and although different plant extract substances diffuse differently in the medium, DDA does not distinguish between the bactericidal and bacteriostatic activity of a compound (Jiang et al., 2013).

In the typical use of DDA for diagnosis, the test aims to guide the correct use of antibiotics, so clinical isolates are used to test paper disks saturated with antibiotics. IZDs are obtained and compared with CLSI Guideline tables, which are used to classify the microorganism as “susceptible”, “intermediate”, or “resistant” to a specific antibiotic, which in turn provides information that is essential to choosing the best-suited antibiotic for that individual. In the present assay, the extracts producing inhibition zones of any size were selected. In the second round of experiments, the extracts were again analyzed using DDA so that IZDs could be determined, and those extracts that did not repeat antimicrobial activity were eliminated. On the other hand, the active extracts were subjected to MBA so that MBCs could be determined.

Dilution methods are suitable to obtain minimum bactericidal and inhibitory activities due to their capacity to quantify the tested compounds or extracts that can dilute in the broth medium, natural products significantly varies (ANVISA, 2022). The test provides a reference value given as the minimum concentration of the substance or extract that inhibits visible bacteria growth, confirmed by a subculture (as in the present study) or by the use of cell viability tetrazolium dyes (Balouiri et al., 2016). MBA was conducted with the selected antimicrobial extracts to obtain MBCs, which were preferred over MICs due to the colorful characteristics of the extracts that do not favor turbidity visual analysis.

MBA is a fast, easy-to-conduct, low-cost methodology applied to obtain MBCs for the selected active extracts. The microscale involved diminishes the chances of contamination, as it allows for the analysis of small amounts of plant extracts. To optimize the costs of the random-chemotaxonomic plant collection strategy, small amounts of plant material are collected in the field, resulting in small amounts of plant extracts, so it is vital to adjust the *in vitro* assay scales to the use of micrograms of plant extracts (Suffredini et al., 2002; Suffredini et al., 2004; Suffredini et al., 2006a; Suffredini et al., 2006b; Younes et al., 2007; Castilho et al., 2013; Camargo and Suffredini, 2014; Castilho et al., 2014; da Silva et al., 2014; Costa et al., 2016; Suffredini et al., 2016; Martins et al., 2019a; Martins et al., 2019b; Camargo et al., 2020; Silva et al., 2020).

Identifying the extract’s active compounds is crucial, and strategies for accelerating this process are also crucial. As the plant extracts are rich mixtures of chemically diverse substances, chromatography techniques are indicated, although not accessible to all labs. Paper and thin-layer chromatography techniques are more accessible. They can be used in bioautography, a technique developed in 1946 by Goodall and Levy (Goodall and Levi, 1946) that combines compound separation or elution obtained by paper (or TLC) chromatography with microbial diffusion assays. Hence, inhibition zones appear over the separated compounds. In the present study, we used three TLC bioautography tests as the drop diffusion bioautography (DiB), unidimensional bioautography (UB), and bidimensional bioautography (BB).

Drop diffusion in bioautography was applied to evaluate the capacity of the extract substances to diffuse from paper disks and the TLC silica gel plate. The comparison fundamentals are based on the fact that a substance can bond differently to the paper disk cellulose or the silicon oxide from the TLC plate. It was observed that some extracts did not diffuse from the silica gel plate as they diffused from the paper disks, diffused from the paper disk, and for that reason they were not submitted to UB and BB tests. We have also analyzed the relationship between IZDs obtained from DDA and DiB assays, so when DDA IZD and DiB IZD have the same value the ratio is 1, meaning that the chemicals diffuse equally from the paper disk and the silica gel. Our results showed that the extracts diffused more from the silica gel than from paper disks. However, we eliminated many extracts that did not diffuse from the TLC silica gel. In the future, we might fractionate using other techniques, such as partition or dereplication.

Most extracts screened for antimicrobial activity were obtained from plants collected using a random or chemosystematic strategy, regardless of their traditional use. Therefore, there was a lack of toxicological information on the plants, and an *Artemia salina* toxicity assay was conducted to fill this gap. The methodology adopted in the present study is in accordance with the 3R principles of replacement, reduction, and refinement (NC3Rs, 2022) that are embedded in the Brazilian legislation related to laboratory animal use regulated by the National Council for the Control of Animal Experimentation (*Conselho Nacional de Controle de Experimentação Animal*—CONCEA) (CONCEA, 2022). Many extracts were considered non-toxic at a concentration of 1 mg/mL. These findings suggest that they could be used as antimicrobial agents without causing significant side effects. The EC50s obtained in the present study support future toxicity experiments involving the influence over *Artemia* development and behavior, and other *in vivo* assays to be undertaken using zebrafish or rodent models.

The series of tests used to screen and analyze the antimicrobial potential of the extracts and their toxicity resulted in there being different results as to the most active extract and could therefore have led to false results. A ranking system based on scores determined according to the *p*-values and the mean rank differences obtained from statistical analyses was developed to avoid errors. For the toxicity assay, a higher score was given to the less toxic extract, the one with the lower EC50. The sum of the scores enabled us to classify the extracts according to their potential to be more active and less toxic, and to achieve our chief goal, which was to determine which extracts ought to be subjected to chemical or *in vivo* analyses.

## Data availability statement

The original contributions presented in the study are included in the article/**Supplementary Material**, further inquiries can be directed to the corresponding author.

## Author contributions

Conceiving, designing, and supervising the experiments: AV and IS. Carrying out the experiments: AC, ER, FM, GK, GM, IS, ID, KM, KP, KB, JPS, JSS, JA, PJ, PB, RM, RP, and YS. Graduate students: AC, ER, KB, KP, JPS, JA, JSS, PB, and SF. Undergraduate students: FM, GK, GM, KM, PJ, RM, RP, and YS. Analysis and interpretation of data: AC, ER, IS, ID, JPS, JA, JSS, KB, KP, PB, RM, RP, and YS. Field expeditions: AV, IS, MP, and SF. Review of the manuscript: all authors. All authors contributed to the article and approved the submitted version.
