# Supplementary material for: Screening Amazon rainforest plant extracts for antimicrobial activity: a 15-year commitment to the Brazilian biodiversity
Source: Front Antibiot. 2023 Jul 12;2:1122400. doi: 10.3389/frabi.2023.1122400 (PMC11732023; doi:10.3389/frabi.2023.1122400)
Supplement: Supplementary file 1 [file DataSheet_1.zip › Suffredini et al., 2023.PDF]

Supplementary Material – Suffredini et al., 2022

1 Supplementary Data

Supplementary Material should be uploaded separately on submission. Please include any supplementary data, figures and/or tables. All supplementary files are deposited to FigShare for permanent storage and receive a DOI.

Supplementary material is not typeset so please ensure that all information is clearly presented, the appropriate caption is included in the file and not in the manuscript, and that the style conforms to the rest of the article. To avoid discrepancies between the published article and the supplementary material, please do not add the title, author list, affiliations or correspondence in the supplementary files.

2 Supplementary Figures and Tables

2.1 Supplementary Figures

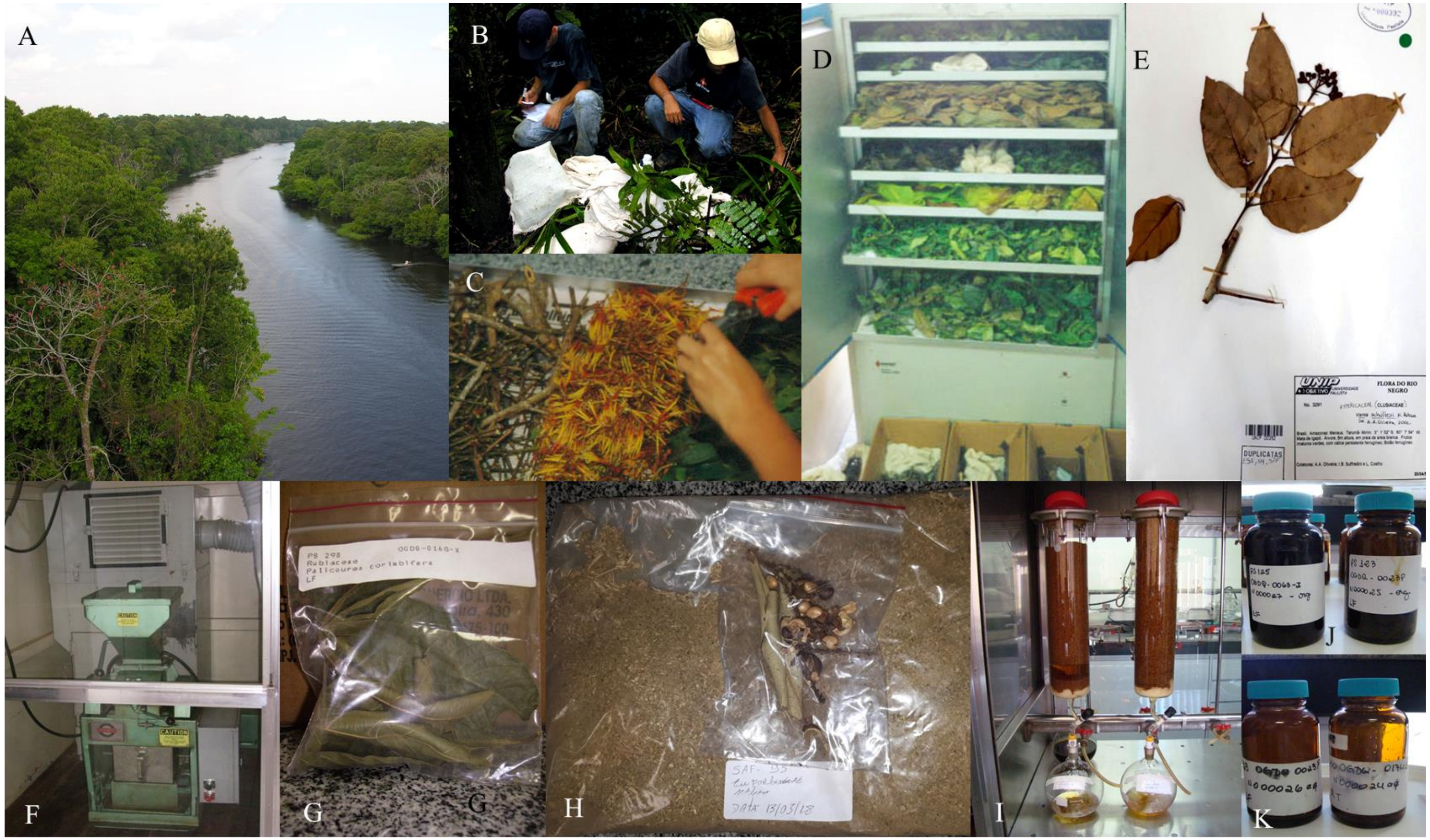

Supplementary Figure 1. Plant collection and extract preparation. **(A).** Igapó forest at the Amazon rain forest; **(B)**. Cleaning plant material in the field; **(C)**. Separating diferente plant organs; **(D).** Plant crude matrial in air-circulating incubator for drying; **(E).** Plant voucher to be added to

the Herbarium; **(F).** Hammer mil to grind dry plant material; **(G).** Voucher of plant material submitted to the grinding process; **(H).** Grouded plant material; **(I).** Ground plant material in maceration;. **(J).** Organic and **(K).** aqueous extracts ready to be frozen at – 20 ^{o}C.

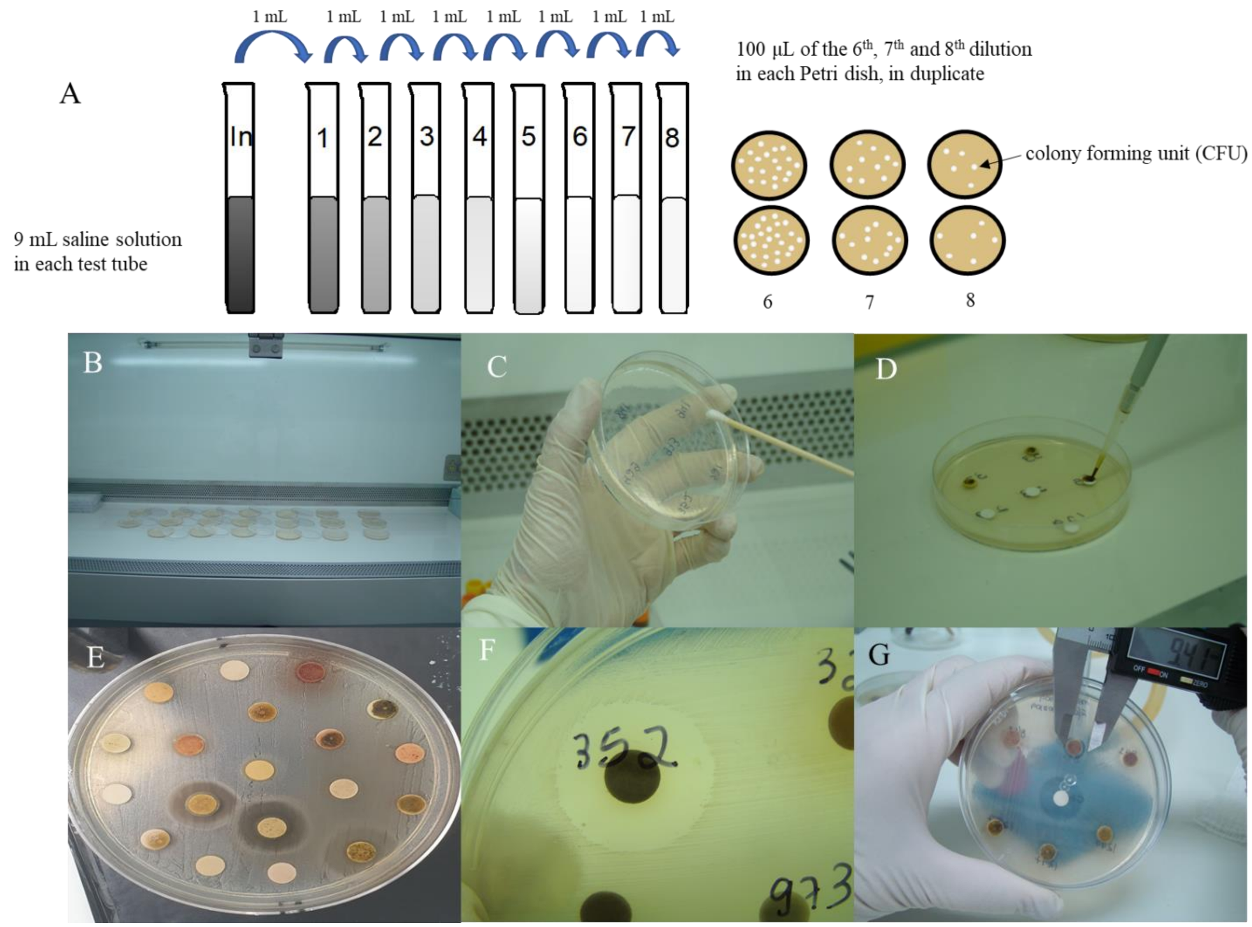

Supplementary Figure 2. Disk diffusion assay. (**A**). Serial dilution at 1/10-fold ratio; **(B).** Petri dish preparation; **(C).** Surface inoculation of the microorganism; **(D).** Extract addition to the sterile paper disk; **(E).** Petri dishes according to the disk distribution at the end of the screening procedure, and inhibition zones; **(F).** Identificatin of each plant extract in the Petri dish; **(G).** Diameter of the inhibition zone being measured with the caliper rule.

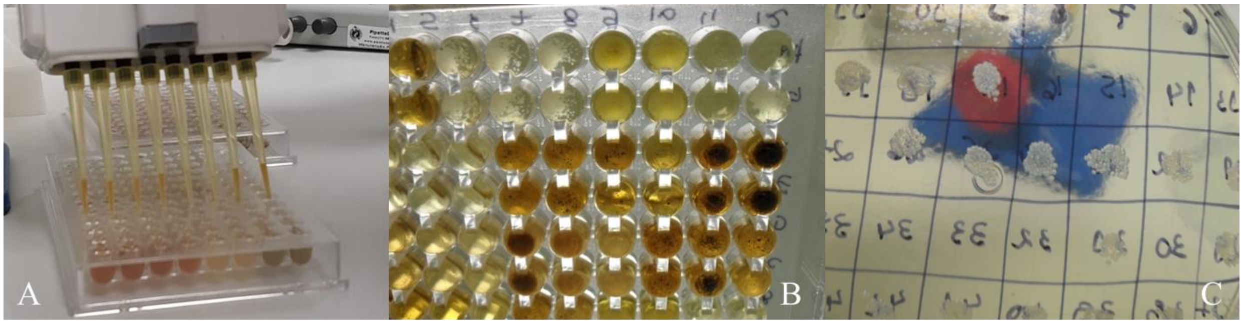

Supplementary Figure 3. Microdilution broth assay. **(A)**. Treatment addition on inoculated medium. **(B).** Medium trubidity scale after incubation. **(C).** Subculture made from each well.

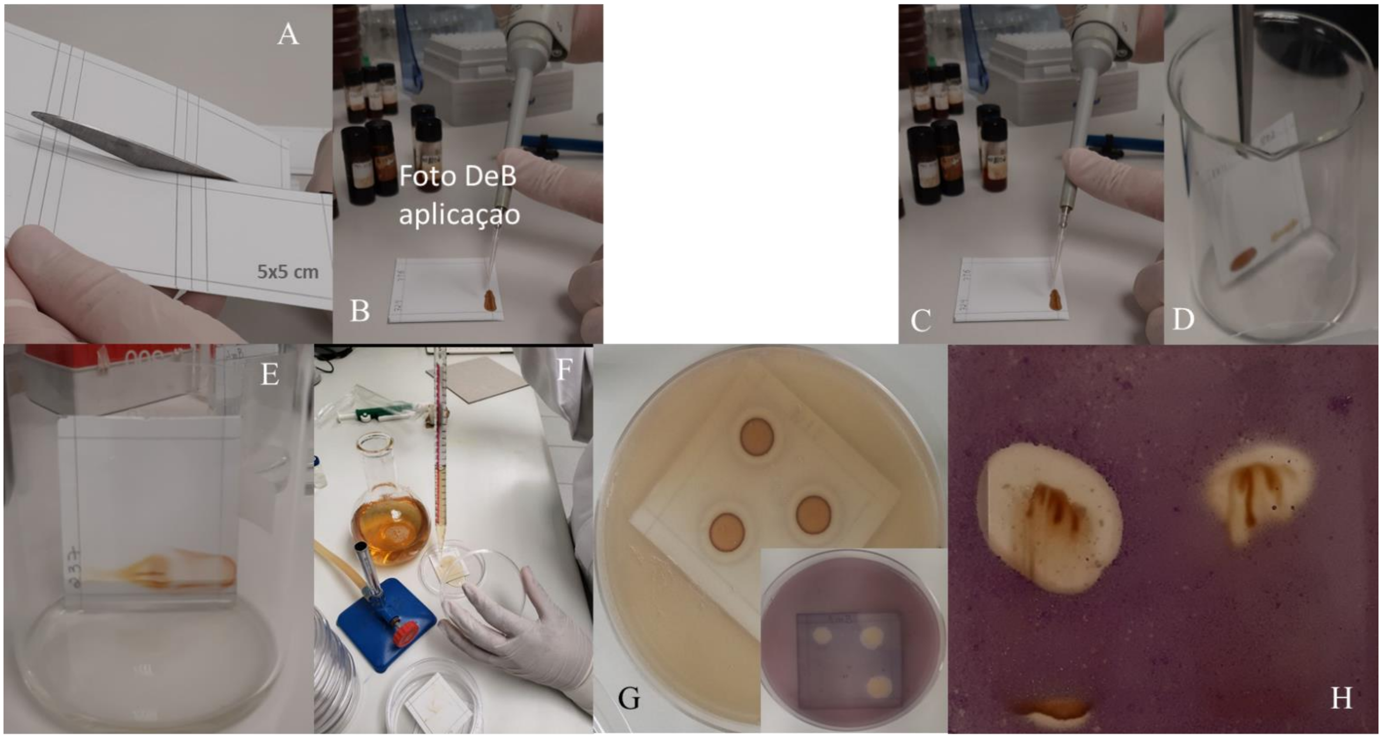

Supplementary Figure 4. Bioautography techniques. **(A).** Thin layer chromatography plates being cut to 5 x 5 cm. **(B).** Drop diffusion in bioautography sample application. **(C).** Unidimensional bioautography sample application. **(D).** Unidimensional bioautography elution in a beaker. **(E).** Bidimensional bioautography elution in a beaker. **(F).** Inoculated medium application over TLC chromatogram. **(G).** Drop diffusion bioautography MTT revelation. **(H).** Unidimensional bioautography MTT revelation.

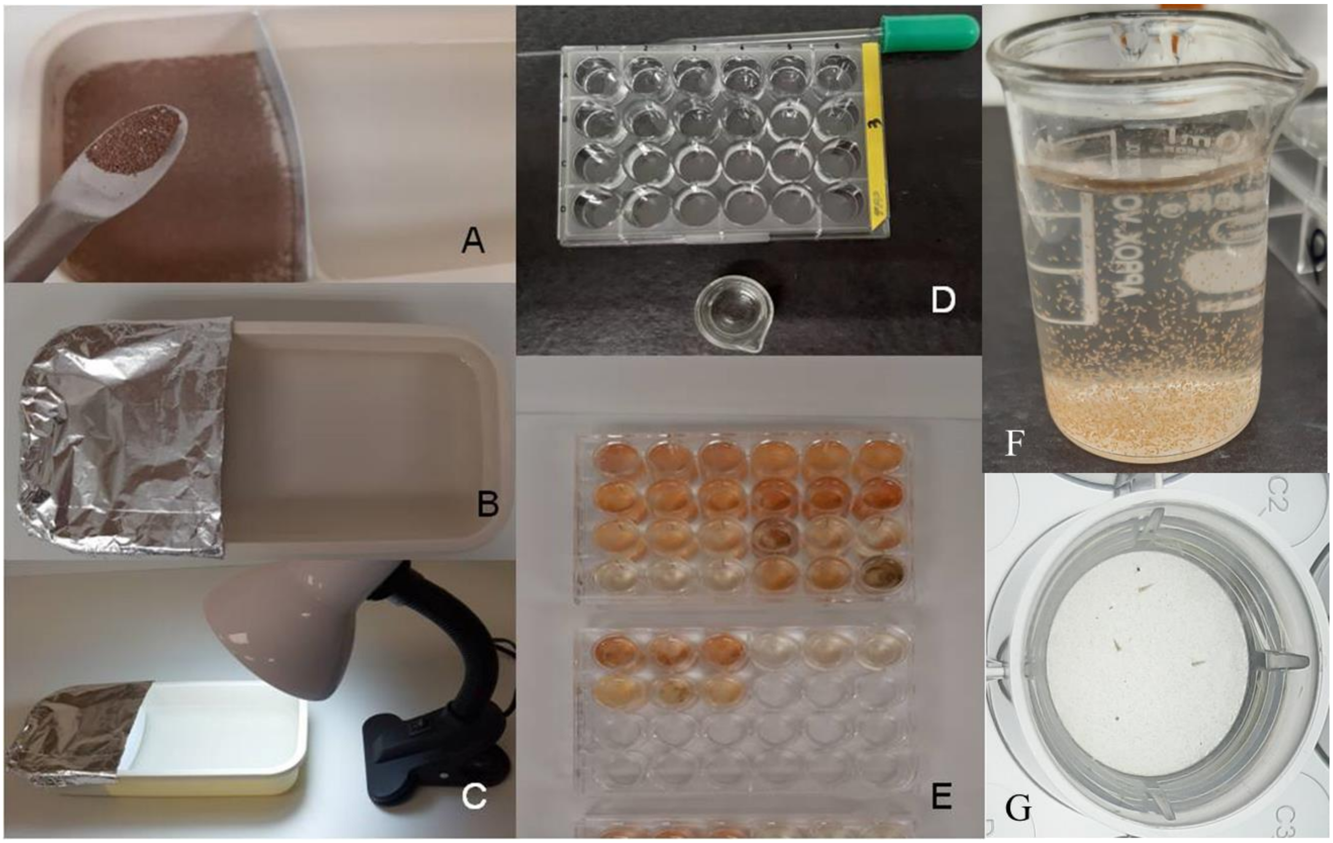

Supplementary Figure 5. Artemia salina assay. **(A).** Aquarium to grow artemia in the adequate compartment; **(B).** Artemia cyst compartment protected from light; **(C).** Light over open compartment to attract nauplii; **(D).** Nauplii being transferred to 24-microplate wells containing marine water; **(E).** Plates after adding treatments; **(F).** Artemia salina 24-h hatched nauplii; **(G).** Nauplii at the end of the experiment.
